# Genipin versus Ferric Chloride cross-linked unmodified Gum Arabic/Chitosan/nano-Hydroxyapatite nanocomposite hydrogels as potential scaffolds for bone regeneration

**DOI:** 10.1038/s41598-023-41413-w

**Published:** 2023-09-01

**Authors:** Lara E. Makar, Norhan Nady, Neivin Shawky, Sherif H. Kandil

**Affiliations:** 1 Department of Materials Science, Institute of Graduate Studies and Research, Alexandria University, El-Shatby, Alexandria, 21526 Egypt; 2 Polymeric Research Department, Advanced Technology and New Materials Research Institute (ATNMRI), City of Scientific Research and Technological Applications (SRTA-City), Alexandria, 21934 Egypt; 3 Oral and Maxillofacial Surgery Department, Faculty of Dentistry, Alexandria University, Champollion Street – Azarita, Alexandria, 21526 Egypt

**Keywords:** Medical research, Materials science, Biomaterials

## Abstract

Ferric chloride (FeCl_3_) and Genipin were utilized as cross-linkers to create two types of nanocomposite hydrogels through physical and covalent cross-linking methods, respectively. The hydrogels were composed of unmodified Gum Arabic (GA), Chitosan (Ch), and natural nano-Hydroxyapatite (nHA) using an acrylic acid solvent. Both the natural nHA and the FeCl_3_ vs. genipin cross-linked GA/Ch/nHA nano-composite hydrogels were prepared and characterized using various in vitro and in vivo analysis techniques. The use of FeCl_3_ and genipin cross-linkers resulted in the formation of novel hydrogels with compressive strengths of (15.43–22.20 MPa), which are comparable to those of natural cortical bone*. *In vivo evaluation was conducted by creating calvarial defects (6 mm) in Sprague–Dawley male rats. The results showed the formation of new, full-thickness bone at the implantation sites in all groups, as evidenced by digital planar tomography and histological staining with Hematoxylin and Eosin stain (H & E). Additionally, the use of genipin as a cross-linker positively affected the hydrogel's hydrophilicity and porosity. These findings justify further investigation into the potential of these nanocomposite hydrogels for bone regeneration applications.

## Introduction

Tissue engineering aims to address the increasing demand for tissue/organ transplants by providing a suitable surrogate matrix that promotes the regeneration of damaged tissues. Making duplicates of native tissue that can serve as substitutes and encourage regeneration is the goal of tissue engineering. In the realm of tissue engineering, three-dimensional (3D) scaffolding is essential. These scaffolds must be porous to allow for appropriate oxygen and nutrient transmission in addition to serving as templates on which new tissue can be grown^1^. Success of a scaffold at its site of action is also determined by mechanical qualities like compressive strength and stiffness, particularly in the regeneration of hard tissue like bone. Natural or synthetic polymers can be used to make the scaffolds, with the former being preferred for its low toxicity^1^. In regenerative medicine, the usage of polysaccharides like chitosan and Gum Arabic is becoming more prevalent^2^.

Chitosan (Ch) is a polysaccharide that is commonly used in regenerative medicine. It has been widely utilized because of its biocompatibility, bioactivity, hydrophilicity, biodegradability, and structural resemblance to glycosaminoglycans (GAGs) found in the extracellular matrix (ECM)^3^. The formation of chitosan hydrogels mainly relies on chemical cross-linking mechanisms. Chitosan is a linear polysaccharide composed of N-acetyl glucosamine (GlcNAc) and glucosamine (GlcN) units, and the primary amine groups on chitosan can be cross-linked through covalent bonds with various cross-linking agents such as glutaraldehyde, genipin, and epichlorohydrin. Moreover, chitosan hydrogels can also be formed through physical cross-linking mechanisms, such as electrostatic interactions with negatively charged molecules or by forming interpenetrating polymer networks (IPN) with other polymers, to improve mechanical properties^4^.

Gum arabic (GA) is an additional naturally occurring polysaccharide that comes from the acacia tree and is non-toxic, biocompatible, and bio-sourced^5^. The United States Food and Drug Administration (USFDA) has given it the designation "Generally Recognized as Safe (GRAS)"^3^. It has been found, however, that GA cannot make hydrogel from this fundamental source material. A potential solution to this issue was the synthetic modification of GA by interaction with glycidyl methacrylate (GMA). After the reaction, GMA altered GA by adding vinyl groups to the polysaccharide structure of GA, enabling the use of hydrophilic vinyl monomers in polymerization reactions. Currently, GA hydrogels treated with vinyl groups are frequently employed to make hydrogels^6^. However, it takes a lot of time and effort to transform GA into modified GA that has been customized with vinyl. Both acrylic acid (AA) and glycolic acid include carboxylic groups that, upon deprotonation, physically cross-link with Fe^3+^. As a result, no chemical changes are made when using GA with the AA monomer. The deprotonated functional groups of AA (COO) and GA (COO and CO) are used to physically cross-link the AA and GA through FeCl_3_·6H_2_O^2,7^. In summary, the gelation properties and mechanisms of GA and chitosan hydrogels differ, with GA relying on physical cross-linking and chitosan on chemical cross-linking. Nevertheless, both hydrogels can be customized for specific applications by adjusting various parameters such as molecular weight, degree of branching, and cross-linking density^4^.

**Figure 1:**
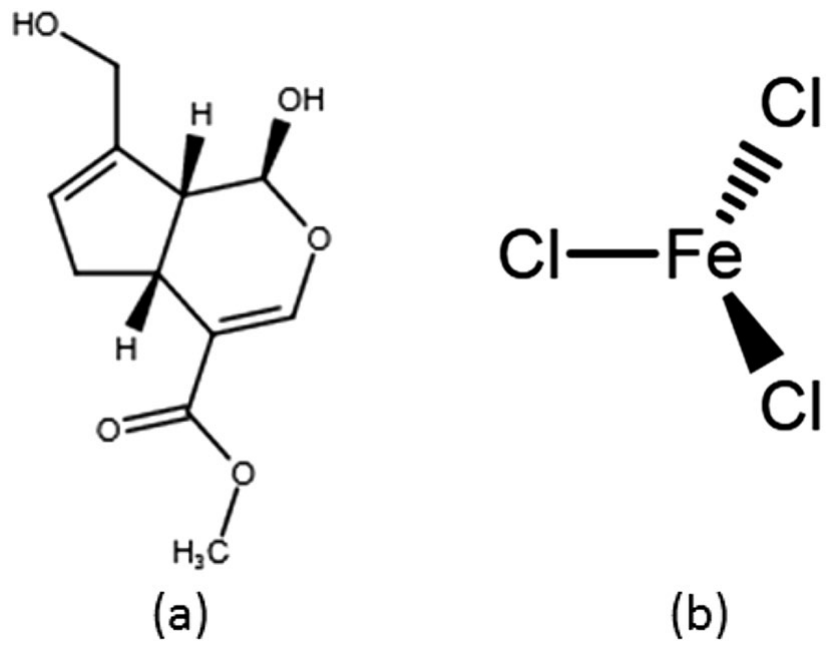
The chemical structure of Genipin (**a**) and FeCl_3_ (**b**) cross-linkers**.**

Chemical cross-linking procedures often use glutaraldehyde, which can produce strongly cross-linked materials in a short time, as seen in several studies^8–10^. Also, it can improve mechanical properties, but has been associated with cytotoxicity due to crosslinker residues which require strict removal processes^10^. Therefore, Genipin (Fig. 1a), a natural cross-linker derived from the fruits of the Gardenial jasminoides Ellis plant, might replace glutaraldehyde because of its low cytotoxicity and antibacterial qualities, which eliminate the possibility of bacterial adherence^11^. In addition to being gastro-, hepato-, and neuroprotective, genipin has choleretic, anti-depressant, antidiabetic, anticancer, antithrombotic and anti-inflammatory^12^. Additionally, it can interact as a bi-functional cross-linking chemical with proteins or amines in general as well as the main amine groups in chitosan to produce flourescent hydrogels that are blue in color and have improved mechanical performance^13^. Additionally, Genipin has a long history of usage in the cross-linking of biomedical implants due to its natural origin, superior biocompatibility, and high cross-linking effectiveness^14^. Chemically cross-linked hydrogels therefore have a number of benefits, such as high selectivity, quick gel formation, and adjustable mechanical characteristics^13^.

On the other hand, the ability of the physical cross-linking approach, to self-heal, which allows the gel network to be destroyed at high shearing forces and then repaired once the shearing forces are gone, is one of its most important characteristic in addition to having adjustable mechanical properties^13^. An example of a physical cross-linker is Ferric chloride (FeCl_3_) as shown in Fig. 1b. In previous work, 2 wt% FeCl_3_ was used to prepare nanocomposite hydrogels that had tunable compressive strengths in the range of 15–22 MPa^2^.

Several approaches to improve the hydrogels' mechanical characteristics are known, such as producing hydrogel composites that combine the structural support provided by the filler materials with the biocompatibility and flexibility of the polymeric network. Bioceramics (hydroxyapatite, tricalcium phosphate), bioglass particles, and carbon nanotubes are the most common fillers employed, allowing the scaffolds used in bone tissue engineering to give temporary mechanical integrity at the defect location as soon as they are implanted^2^. So**,** since natural bone is made up of carbonate-substituted hydroxyapatite (nHA) nanocrystals, the most promising and frequently chosen method for the production of bone tissue scaffolds involves combining nHA fillers with natural polymers. The nHA provides a source of calcium and phosphate ions, that are essential for cell survival while also being osteoconductive and bioactive^12,15^.

This study aimed to compare the use of Ferric chloride physical cross-linker and Genipin chemical cross-linker in the preparation of unmodified Gum arabic/Chitosan/nanoHydroxyapatite (GA/Ch/nHA) novel nanocomposite hydrogels to analyze their potential use in bone regeneration. Addition polymerization gelation reaction was used, and a combination of 15% GA/Ch and 5% nHA concentrations were chosen as they had good properties and performance as revealed from our previous study^2^. The Genipin chemical cross-linker concentration of 0.5% w/w was determined as 1% w/w genipin was found difficult to prepare in the laboratory as it had high viscosity with difficult mixing. The prepared hydrogels were characterized using different analysis techniques such as Fourier transform infrared spectroscopy (FT-IR) analysis, X-ray diffraction (XRD) analysis, energy dispersive X-ray (EDX), and scanning electron microscopy (SEM) imaging, thermogravimetric analysis (TGA) and differential scanning calorimetry (DSC). In addition, the hydrogels were studied for their morphology, mechanical properties, in vitro degradation, apatite forming ability, and in vivo bone regeneration capability in the calvarial defects of Sprague–Dawley rats.

## Materials and methods

### Materials

Acrylic acid (purity > 99%) was purchased from Alpha Chemika, (Mumbai, India). Gum Arabic (GA) (purity > 98%) was obtained from Acros (Geel, Belgium). Chitosan (Mw 100,000–300,000, purity > 99%) was purchased from Bio Basic (Canada Inc). Ferric chloride hexahydrate (FeCl_3_·6H_2_O, purity > 98%) was purchased from El-Nasr Pharmaceutical Chemicals Company (Oubour, Egypt). Genipin was purchased from Acros Organics (Fisher Scientific USA). Ammonium persulfate ((NH_4_)_2_S_2_O_8_ (APS); purity > 99%) was obtained from Biochem laboratory chemicals (Cairo, Egypt). Sodium phosphate dibasic (purity > 99%, Na_2_HPO_4_), Potassium dihydrogen orthophosphate (purity > 98%, KH_2_PO_4_), Sodium Sulphate (purity > 98%, Na_2_SO_4_), Potassium chloride (purity > 99%, KCl), Calcium Chloride anhydrous (purity > 99%, CaCl_2_), and Sodium chloride (purity > 99.5%, NaCl) were obtained from Oxford Lab Chem, (Mumbai, India). Sodium Bicarbonate was purchased from El Gomhouria Co. (Egypt). Magnesium chloride MgCl_2_·6H_2_O was purchased from Nice Chemicals Ltd (Kerala, India).

### Methods

#### Preparation of nano-Hydroxyapatite from natural bovine bone

As stated in our previous study^2^, the bovine bone was washed carefully with water and acetone to remove the fats and other impurities. Then, the bone was dried at 160 °C for 48 h. The cleaned dried bones were annealed in an electric furnace (Nabertherm 30-3000. Germany) at 750 °C for 6 h^16^. The bone converted from a brown to white color. Then, the bone was ground with mortar and pestle to a particle size less than 450 µm.

#### Preparation of unmodified Gum Arabic/Chitosan/nano-Hydroxyapatite nanocomposite hydrogels with ferric chloride and genipin as cross-linkers

**Figure 2:**
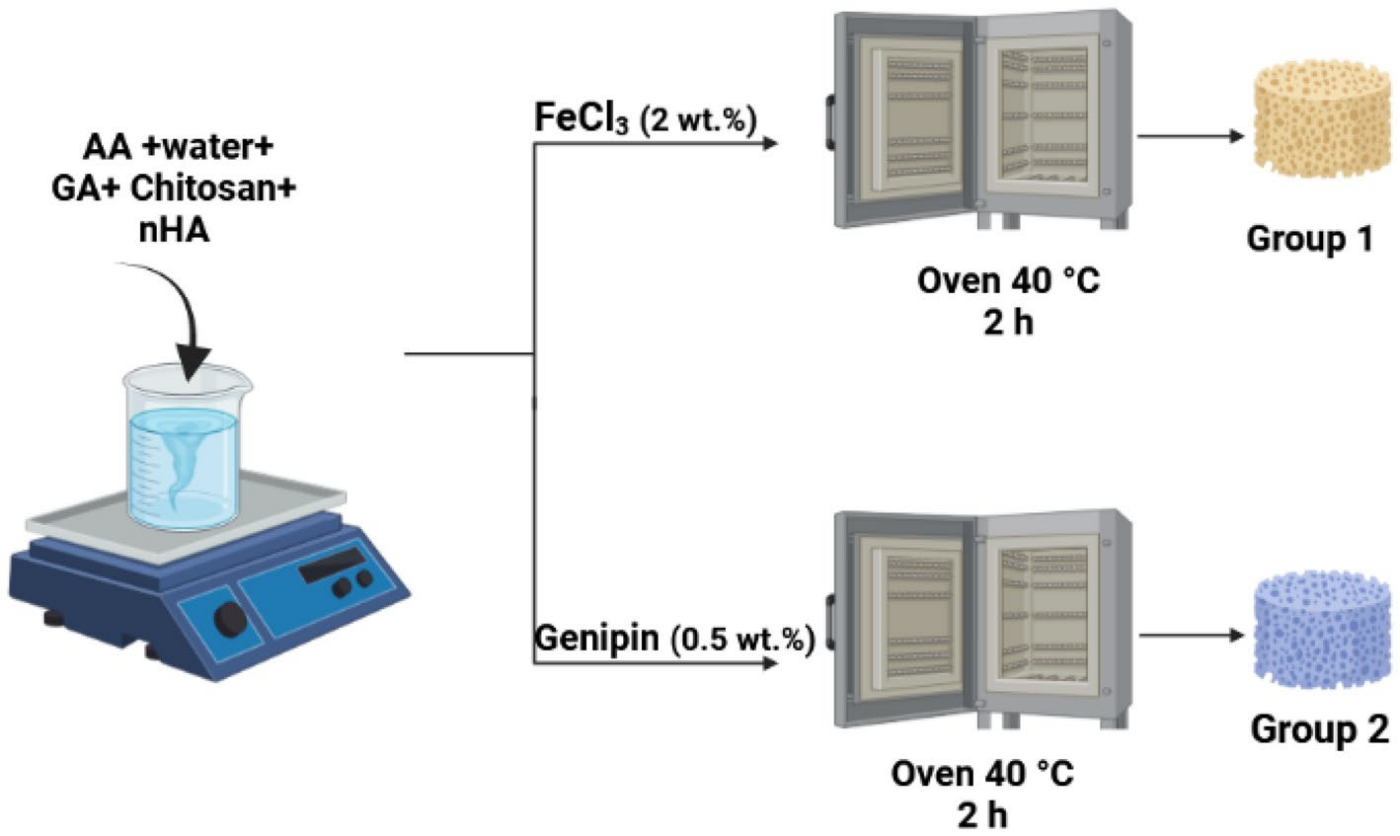
Schematic diagram illustrates the preparation steps of GA/chitosan/nHA hydrogels.

The free radical addition polymerization was used for the physically/chemically cross-linked composite hydrogels as shown in Fig. 2. In a typical procedure, Initially, 4.2 g AA in 10 ml distilled water was prepared in a reaction vessel and then followed by the addition of 0.25 g Chitosan and 0.38 g GA which is 15% of the used AA. The mixture was stirred till the formation of a homogeneous solution. Then, 5% nHA powder was added to the blend with continuous stirring. After that, for Group 1; 2 wt% FeCl_3_ was added to the blend as a physical cross-linker and for Group 2; 0.5 wt% Genipin as a chemical cross-linker. Stirring was continued for both groups for 2 h. Then, 0.1 g of ammonium persulfate was added to the blend. The blend was placed in the oven for 2 h at 40 °C for complete polymerization. Then, the hydrogels were kept in a petri dish for drying at ambient temperature.

#### Characterization of the naturally prepared nano-Hydroxyapatite (nHA) and GA/Chitosan/nHA hydrogels.

##### Energy dispersive X-Ray spectroscopy (EDX)

The elements and chemical groups of the prepared nHA were identified using energy dispersive X-Ray spectroscopy (EDX, JEOL IT200-JSM, Tokyo, Japan). Specimens were investigated at multiple magnifications with SEM, operating at 20 kV accelerating voltage.

##### Scanning electron microscopy (SEM) imaging

Scanning electron microscopy (SEM) (JEOL 5300-JSM, Tokyo, Japan) was used to investigate the microstructure and surface morphology of the GA/chitosan/nHA nanocomposite hydrogels. Prior to SEM, the specimens were mounted on aluminum stubs using conductive tape and sputter-coated with a gold thin layer for 100 s (Jeol Fine Coat Ion Sputter, JFC 1100E, MA, USA). The samples were imaged at a voltage of 20 kV; 100 ×, 1000 ×, 3000 × and 10,000 × magnifications were used.

##### Fourier transform infrared spectroscopy (FTIR) analysis

The Fourier transform infrared spectroscopy (FTIR) spectra for the prepared nHA and GA/chitosan/nHA nanocomposite hydrogels were recorded throughout the wavenumber range from 4000 to 400 cm^−1^, at a resolution of 4 cm^−1^ using the Perkin-Elmer FTIR spectrometer.

##### Mechanical testing

A mechanical measuring system (DMA 7e—Perkin Elmer, Waltham, MA, USA) was used to determine the compressive strength and strain (%) of the GA/chitosan/nHA nanocomposite hydrogels. The cylindrical samples were manufactured with a diameter of 1.5 cm and a thickness of 1 cm. At a strain rate of 0.5 mm/min, a uniaxial compression test was performed.

##### Thermal stability

The thermal stability was studied with thermogravimetric analysis (TGA) and differential scanning calorimetry (DSC), carried out in a nitrogen atmosphere using a Shimadzu TGA-50H and DSC-50 thermal analyzer^1^.

##### X-ray diffraction (XRD) analysis

X-ray diffraction patterns of the prepared nHA, GA/chitosan hydrogel, and GA/chitosan/nHA nanocomposite hydrogel were obtained by a Shimadzu XRD-7000 X-ray (Kyoto, Japan) diffractometer using a CuKα radiation source operating at 40 kV and 30 mA.

##### Water absorption

Water absorption was determined according to ASTM D570. For each hydrogel, three different discs of 5 cm diameter and 0.5 cm thickness were used. They were immersed in water for predetermined short intervals (5, 10, 15, 30, and 60 min) and 2, 6, 12 and 24 h; the samples were removed from the water, wiped down, and weighed to determine their wet weights (W_w_). Then, they were dried in an oven at 40 °C until they reached a consistent weight and the hydrogel discs were weighed (W_d_). Water absorption is expressed using Eq. (1)^17^.

1 $$\mathrm{Water \,\,Absorption }\left(\mathrm{\%}\right)=\frac{\mathrm{Ww }-\mathrm{ Wd}}{\mathrm{Wd}} \times 100$$

##### Degradation studies

Three samples of each GA/chitosan/nHA hydrogel were immersed in phosphate buffered saline (PBS; pH = 7.4) at 37 °C to study the samples’ in vitro degradation. In sterile glass test tubes, pre-weighted (W_i_) dry samples (hydrogels) were soaked in 5 mL PBS and incubated for 56 days (about 2 months). At 14, 28 and 56 days, the samples were removed from the solution, washed with deionized water, dried at 40 °C until the mass remained unchanged, and measured. After each measurement, each respective tube was replenished with fresh PBS. Finally, the samples’ dry weight (W_d_) was recalculated, and the percentage of biodegradation was measured using Eq. (2).

2 $$\mathrm{Degradation} \left(\%\right)=\frac{\mathrm{Wi}-\mathrm{Wd}}{\mathrm{Wd}}\times 100$$

##### Bioactivity evaluation

The simulated body fluid (SBF, 1 L) with pH 7.4 was prepared and if necessary, the pH was adjusted using hydrochloric acid or sodium hydroxide^2,18^. The bioactivity of the GA/chitosan/nHA nanocomposite hydrogels was assessed in vitro in SBF at 37 °C, as described in previous study^19^. The hydrogel samples (0.5 mg) were immersed in SBF (5 mL) for 14 days. At the end of the 14 days, the hydrogels were removed from the SBF and dried at 37 °C. The formation and microstructure of an apatite layer on the surfaces of the hydrogels was evaluated by using SEM and EDX.

##### In vivo bone regeneration of GA/chitosan/nHA nanocomposite hydrogels

*Calvarial defect model* Sterile surgical techniques were applied throughout the experiment. The experimental protocol was approved by the Institutional Animal Care And Use Committee (IACUC), Alexandria University^20^, which comply with the NIH guidelines for the care and use of laboratory animals (NIH Publication #85–23 Rev. 1985)^21^ and follow the recommendations in the ARRIVE guidelines^22^.

**Figure 3:**
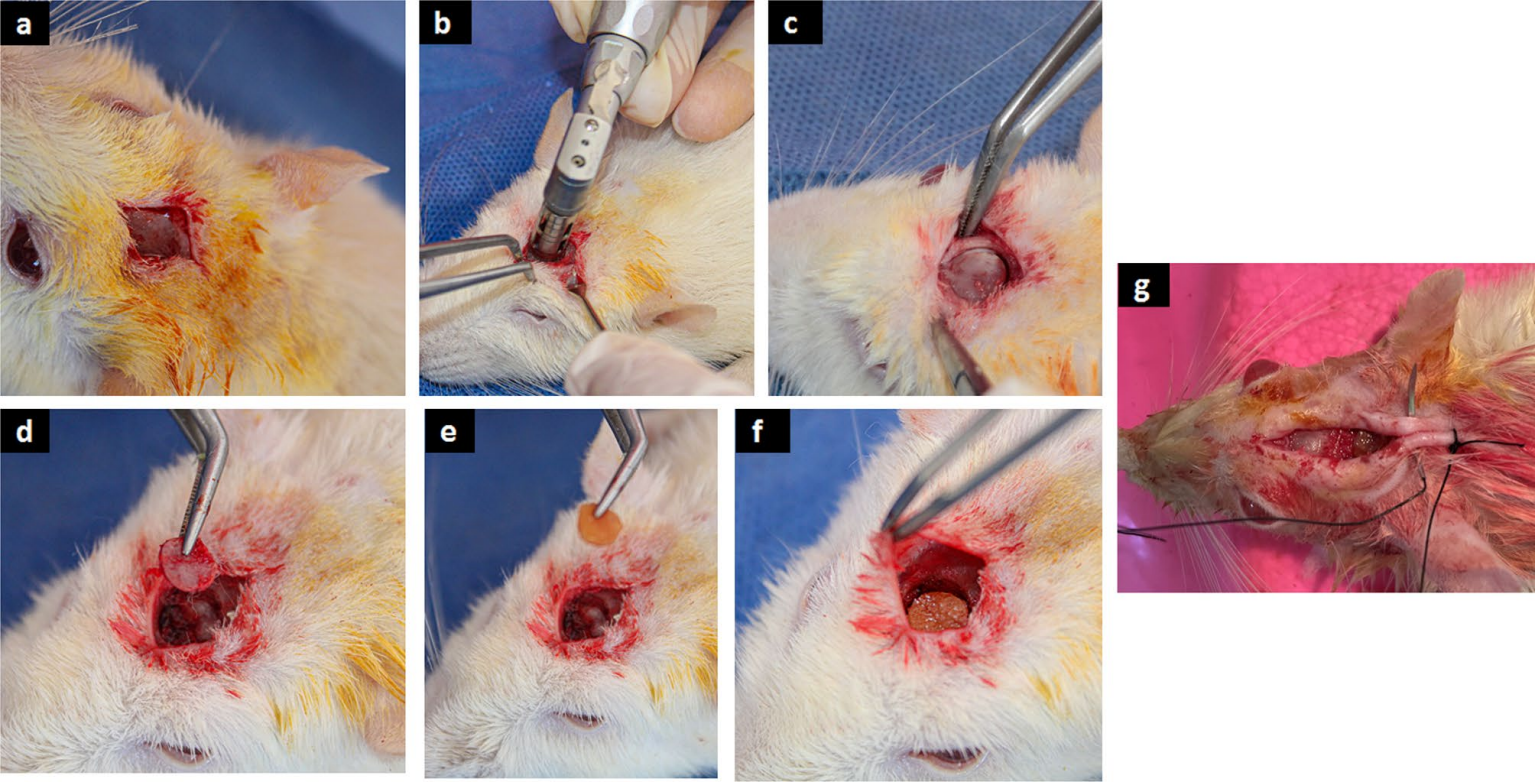
(**a**) Midline incision with skin and periosteum reflection exposing the sagittal suture of the calvarium. (**b**) dental trephine (6 mm diameter) with a dental drill against the superficial aspect of the calvarium. (**c** and **d**) removing the 6 mm bone. (**e** and **f**) inserting the scaffold implant. (**g**) suturing.

Sprague–Dawley (8–12 weeks old) male rats, 300-370 g (n = 15, 5 in each group) were anaesthetized using: Ketamine 50 mg/Kg, xylazine 5 mg/kg^23^. As shown in Fig. 3, all groups underwent the creation of a calvarial defect by using a dental trephine (6 mm diameter) with a dental drill against the superficial aspect of the calvarium. This exposure was achieved through a midline incision and spreading of the skin, fascial and periosteal layers overlying the sagittal suture of the calvarium. The bone was not completely penetrated by the trephine to avoid damage to the underlying dural and brain tissues as the dura plays a role in bone healing and regeneration. Instead the bone was thinned considerably and elevated using blunt instruments to separate the bone from the underlying dura. Once the bone was excised, each defect was syringed with sterile saline solution to remove bone debris and then implanted with FeCl_3_ cross-linked GA/Ch/nHA nanocomposite hydrogels in Group 1; genipin cross-linked GA/Ch/nHA nanocomposite hydrogels in Group 2; and an empty defect as negative control in Group 3*.*

Afterwards, the wound was closed by suturing the periosteam using absorbable 3–0 catgut sutures and next the skin layers were approximated using 3–0 silk sutures. After surgery, all animals were allowed to recover on a warm sheet and then transferred to the vivarium for postoperative care. Postoperatively each rat received antibiotic (ceftriaxone, 5 mg/kg and antibiotic ointment over the shaved skin) and anti-inflammatory (ketofan 5 mg/kg) for 3 days^24,25^.

*Digital planar tomography *Four weeks post implantation, animals were euthanized and calvarial tissues were harvested for analysis. This agrees with previous studies that state that rats can be euthanized from 4 to 12 weeks to evaluate bone regeneration^25,26^. The extracted calvarial tissues were fixed in 4% formaldehyde at room temperature. After 48 h, the fixed samples were rinsed with PBS and imaged using planar digital radiography (RVG 142) for 10 s at 25 kVp and 3 mA. The new bone surface area was measured using ImageJ software (NIH, Bethesda, MD), and normalized to the original defect surface area (6 mm in diameter)^25^.

*Histological evaluation *The fixed tissues were decalcified in 10% EDTA solution under gentle shaking for 1 week. The EDTA solution was changed once on the third day. Decalcified samples were embedded in paraffin and cut into 5-μm thick sections. The tissue sections were deparaffinized and stained with H&E^24^.

## Results and discussion

### Characterization of the natural NanoHydroxyapatite (nHA)

The nanoHydroxyapatite (nHA) prepared and used in previous work^2^, was used in this work also. It was previously characterized in which the Energy Dispersive X-Ray Spectroscopy (EDX) analysis showed evidence of phosphorus, oxygen, and calcium with the calcium/phosphorus atomic ratio (Ca/P) of 1.73, which is essential to keep the mechanical properties of the nHA^27^. Also, the morphological analyses of the nHA that was extracted from the bovine bone revealed particles that are mostly irregular in shape with the presence of rods, flakes, needles, and plate-like shapes. Moreover, the XRD patterns of the nHA were consistent with the crystallinity of the HA results (JCPDS-PDF no. 74-0566)^28^.

**Figure 4:**
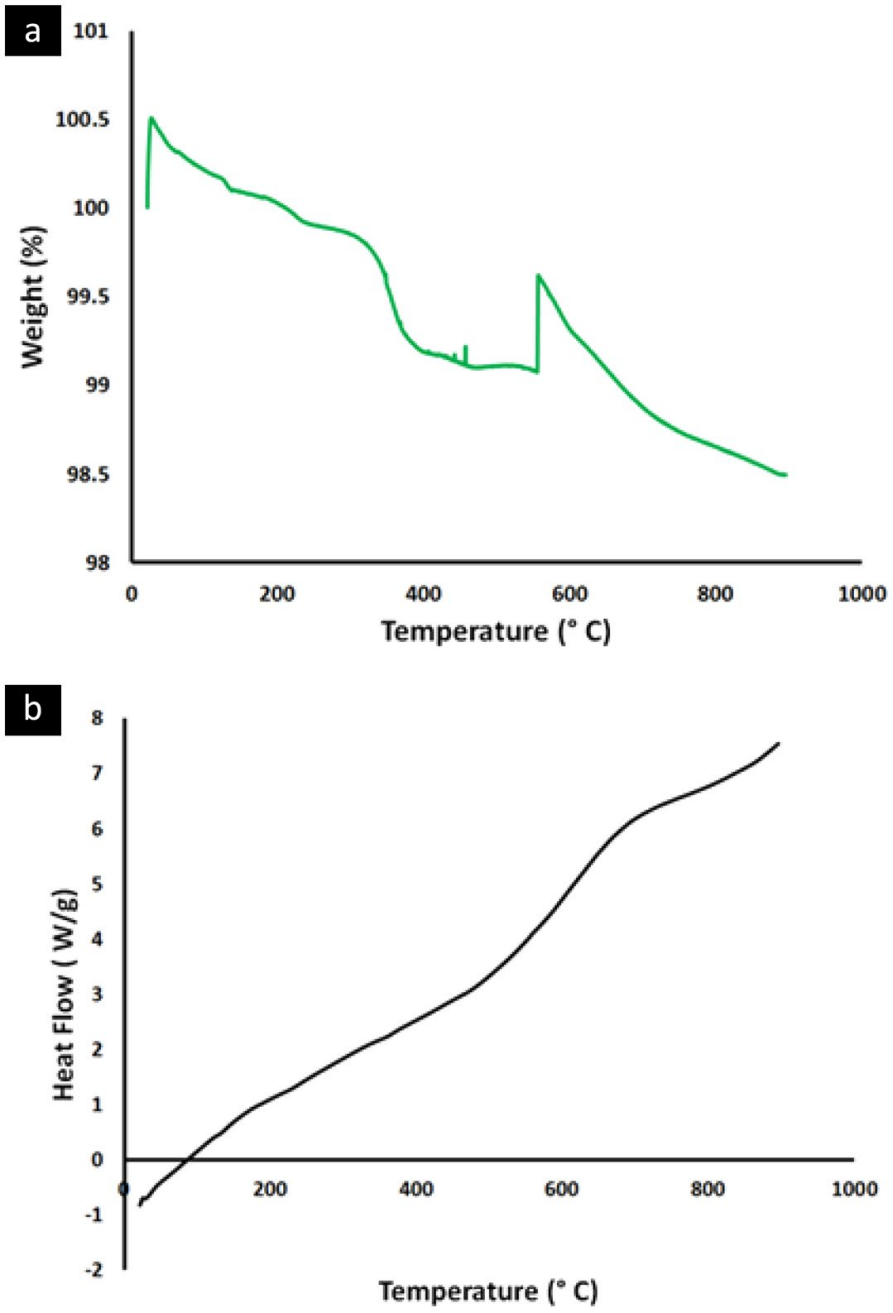
Thermal stability analysis for the prepared natural nano-Hydroxyapatite (nHA) powder; (**a**) thermogravimetric analysis (TGA) (**b**) Differential scanning calorimetry (DSC) analysis.

In addition, further thermal stability analyses were performed; The thermogravimetric analysis (TGA) results of the prepared nHA are shown in Fig. 4a. The TGA curves display a continuous slow mass loss with a constant temperature increase. The peak observed at less than 100 °C indicates weak absorbed moisture loss. At about 100 to 150 °C, there is a small slope change in the TGA curve, which can be attributed to the loss of strongly adsorbed water whose loss rate is rather low and continues to end at around 300 °C. A fast slope change of the TGA curve is shown around 300–400 °C which can be attributed to the dehydroxylation of the nHA. Then a small peak at about 420 °C that may be related to the decomposition of the carbonate ions was detected by the FTIR spectrum in the previous work^2^ and can be attributed to the absorbed carbon dioxide by the nHA. Finally, another sudden change in the TGA curve can be seen at about 600 °C that can be attributed to phosphate ion decomposition. These results indicated the stability of the prepared nHA at temperatures around 600 °C^29^.

Differential scanning calorimetry (DSC) data for nHA in Fig. 4b, was rather featureless, revealing only a broad endotherm centred at 85 °C, which is typical when water molecules are removed^29^. Another exothermic peak was identified at around 750 °C that can be attributed to the rearrangement of the CO_3_^2-^ and HPO_4_^-^ groups present in nHA. This is in full agreement with the TGA analysis.

### Characterization of the GA/Ch/nHA hydrogels

#### Fourier transform infrared spectroscopy (FTIR) analysis

**Figure 5:**
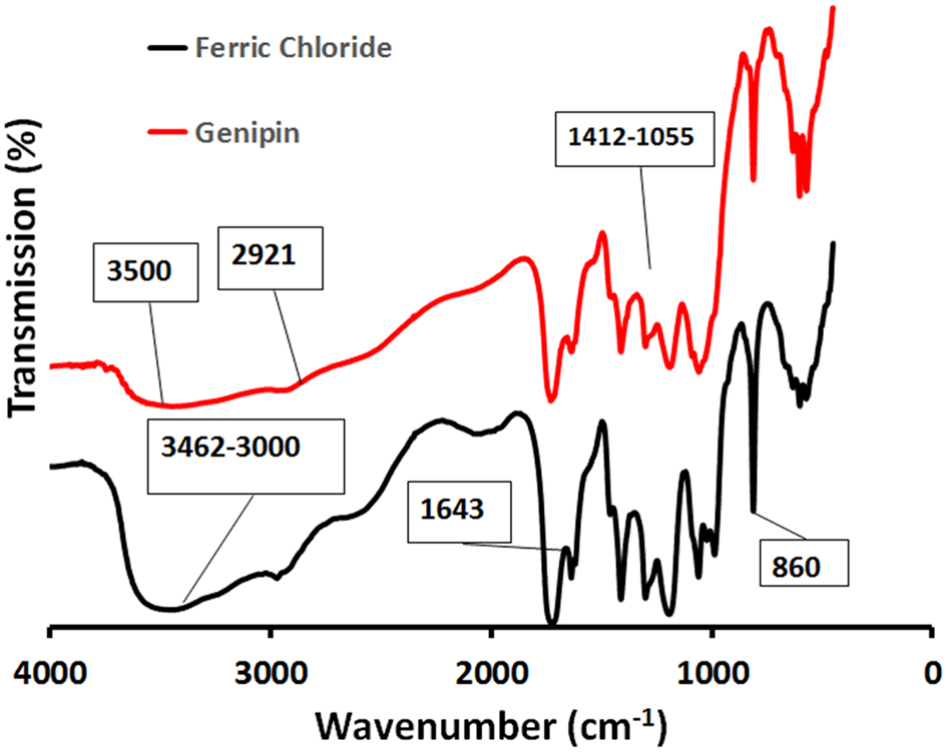
The FTIR spectra of the prepared FeCl_3_ and Genipin cross-linked GA/Ch/nHA hydrogels.

Figure 5 shows the Fourier Transform Infrared Spectroscopy (FTIR) spectrum for the 15% GA/Ch/nHA hydrogels with the two distinct cross-linkers; FeCl_3_ and Genipin, respectively. As shown in the figure, the FeCl_3_ cross-linked hydrogel has chitosan characteristic absorption bands at 3462–3000 cm^–1^ that can be attributed to the primary amine (NH_2_) and the (OH) group associated with the pyranose hydroxyl group. The amide band (C=O) in NHCOCH_3_ groups resonate in the spectrum at 1643 cm^–1^. The CH_3_COH group in pyranose ring vibration has bands at about 860 cm^–1^, which correspond to the saccharide structure. Moreover, GA has characteristic absorption bands at 3413 cm^–1^for O–H stretching, characteristic of the glucosidic ring, and 1200–900 cm^–1^which is the fingerprint of carbohydrates^3^. FTIR of Genipin cross-linked hydrogels shows the typical OH stretch at 3500 cm^–1^, with C–H vibrations at 2921 cm^–1^. An increase in the number of bands between 1412 and 1055 cm^–1^, indicating C–N stretch and C–O–H vibrations, is observed after Genipin cross-linking^1^

#### Morphological analyses of GA/Ch/nHA nanocomposite hydrogels

**Figure 6:**
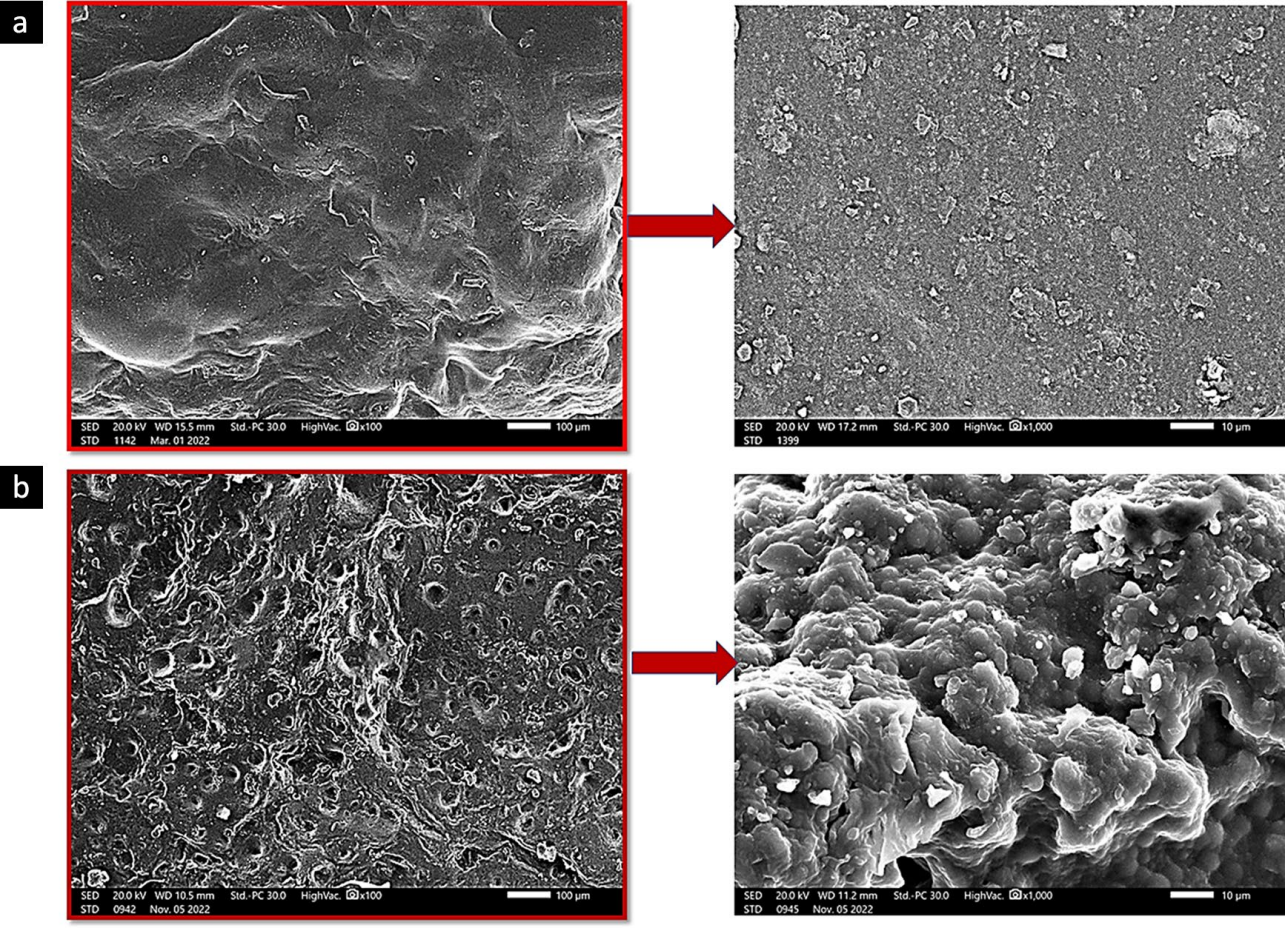
SEM images of the prepared GA/Ch /nHA hydrogels; (**a**) GA/Ch/nHA using FeCl_3_ cross-linker, (**b**) GA/Ch/nHA using Genipin cross-linker.

The SEM images in Fig. 6 demonstrate the hydrogels' excellent cross-linking capacity. This is evident by the compactness of the hydrogels' surface within the gel network. Additionally, the nHA uniform dispersion is discernible^9^. Also, revealed in Fig. 6b is that Genipin induced the corrugation of the scaffold surface and the emergence of more small interconnected pores. These results are in agreement with Zhenchao Guo et al.^30^ that stated that an increase in Genipin concentration increases the scaffold’s porosity. These pores could encourage cell adhesion and aggregation as well as ingrowth along the pores of scaffolds.

#### Mechanical properties

**Figure 7:**
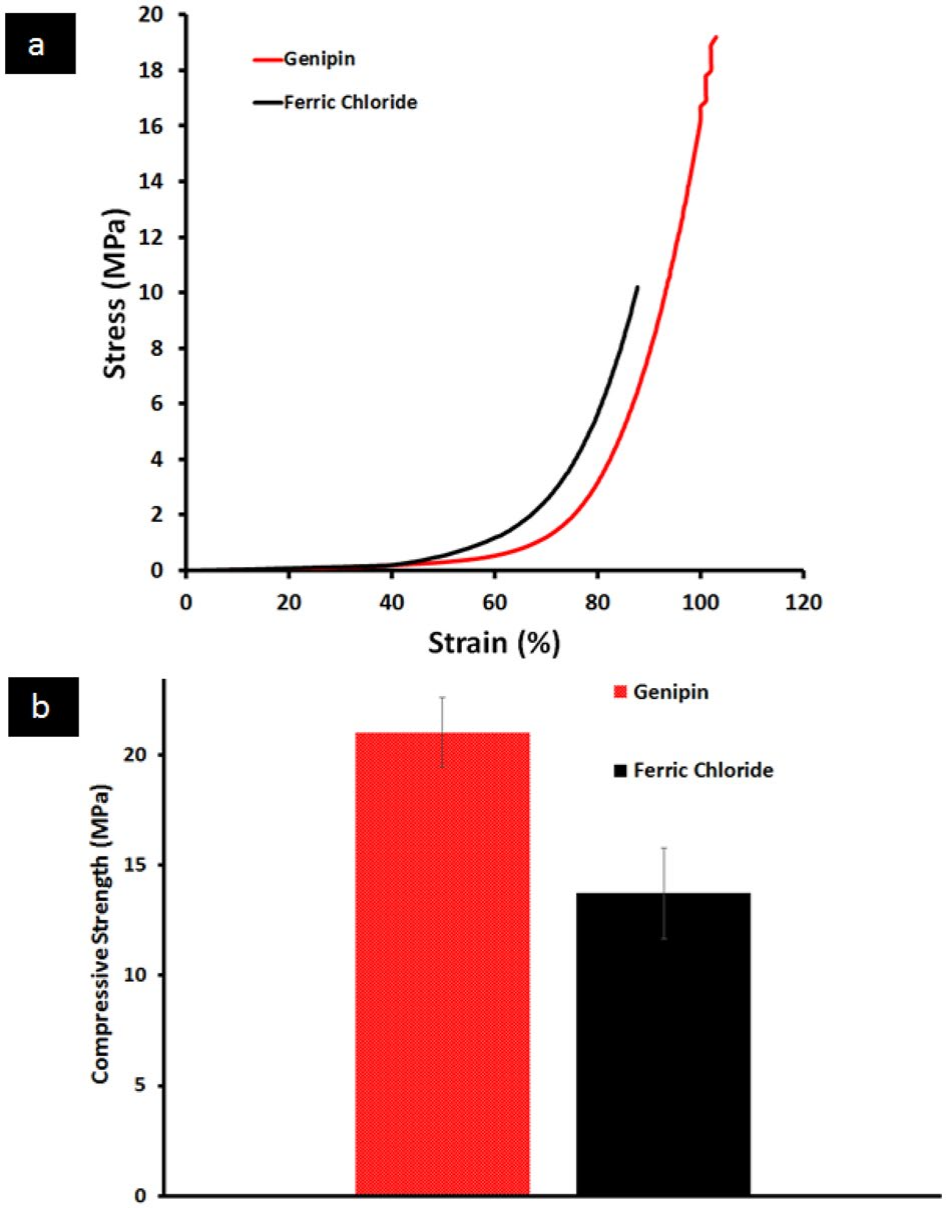
(**a**) Stress–strain and (**b**) compressive strength plot of the prepared GA/Ch/nHA hydrogels prepared using FeCl_3_ and Genipin cross-linker.

The compressive strengths of the prepared FeCl_3_ and Genipin cross-linked GA/Ch/nHA hydrogels are shown in Fig. 7. The compressive strengths of all the prepared hydrogels ranged from 10.2 to 19.2 MPa on average, which is comparable to the range of compact alveolar bone, which is 1–100 MPa.^31^. The compressive strengths were 13.7 ± 2.1 MPa and 21 ± 1.57 MPa for FeCl_3_ and Genipin cross-linked GA/Ch /nHA hydrogels respectively. Using a Genipin cross-linker instead of a FeCl_3_ cross-linker improved the compressive strength of the hydrogel by around 61%. This can be attributed to the bi-functional cross-linking nature of genipin with the primary amine groups in chitosan^13^. Primarily, hydrogels used to guide tissue regeneration, must exhibit biomechanical properties that largely mimic those of the native tissue. In this context, we hypothesized that the addition of Genipin cross-linker could be a way to improve its mechanical properties and thus enhance bone regeneration.

#### Thermal stability; thermogravimetric analysis (TGA) and differential scanning calorimetry (DSC)

**Figure 8:**
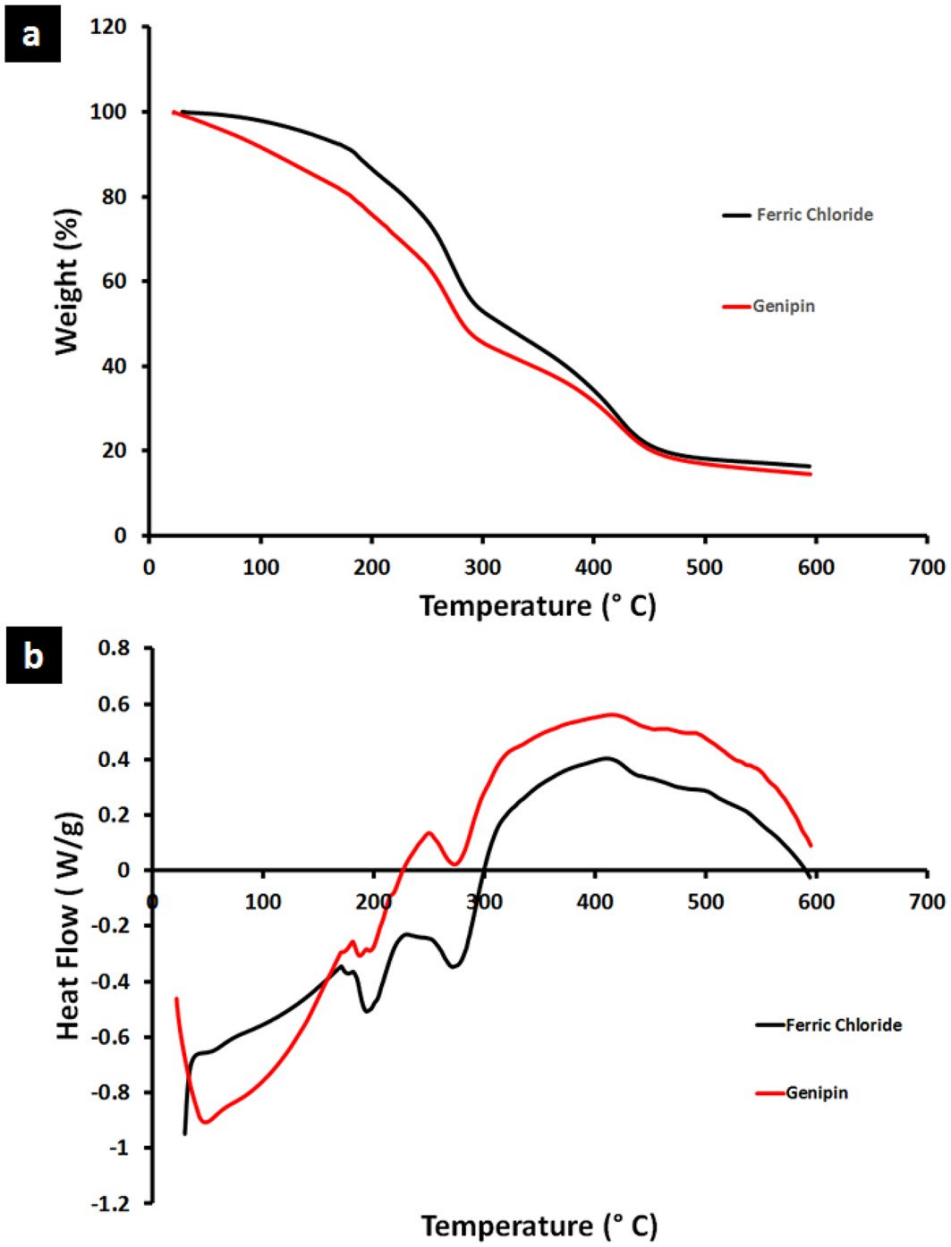
Thermal analysis of the prepared GA/Ch/nHA) hydrogels using FeCl_3_ and Genipin cross-linkers; (**a**) The thermogravimetric analysis (TGA) and (**b**) Differential scanning calorimetry (DSC) analysis.

Thermal properties of FeCl_3_ cross-linked and Genipin cross-linked hydrogels were evaluated using TGA under a nitrogen atmosphere as shown in Fig. 8a. From TGA analysis of FeCl_3_ cross-linked hydrogel, the first weight loss step that occurred around 100–150 °C may be attributed to the loss of residual water. The second stage of weight loss occurred at around 200–280 °C can be attributed to decomposition of Gum arabic^32^ with a weight loss of around 33%. The third region occurred around 280–450 °C can be attributed to the decomposition of chitosan^33^ with weight loss of around 24%. Finally, the fourth region around 450–600 °C, may be due to the very slow degradation of residual complex, phosphate ions and nHA^32^.

TGA of Genipin cross-linked hydrogels shows two significant weight losses, one weight loss at 180–277 °C of 28% due to moisture vaporization and the other at 312–420 °C which was due to the thermal degradation of chitosan which showed a weight loss of 17%^32^.

The differential scanning calorimetry (DSC) thermogram in Fig. 8b shows FeCl_3_ cross-linked hydrogel having a sharp endothermic peak at 194 °C and 274 °C, which can be attributed to the loss of water from the sample; this is a non-equilibrium process, and a second thermal event that was revealed by a broad exothermic peak, which can be found at 298 °C and is attributed to the decomposition of glucosamine units in chitosan. When chitosan is cross-linked with Genipin, the endothermic peak shifts to 49 °C and 274 °C while the exothermic peak shifts to 286 °C. Generally, cross-linking results in an increase in thermal stability; however, other researchers agree that the incorporation of Genipin leads to an increase in hydrophilicity of the network and therefore results in a lower decomposition temperature^1^.

#### X-ray diffraction (XRD) analysis

**Figure 9:**
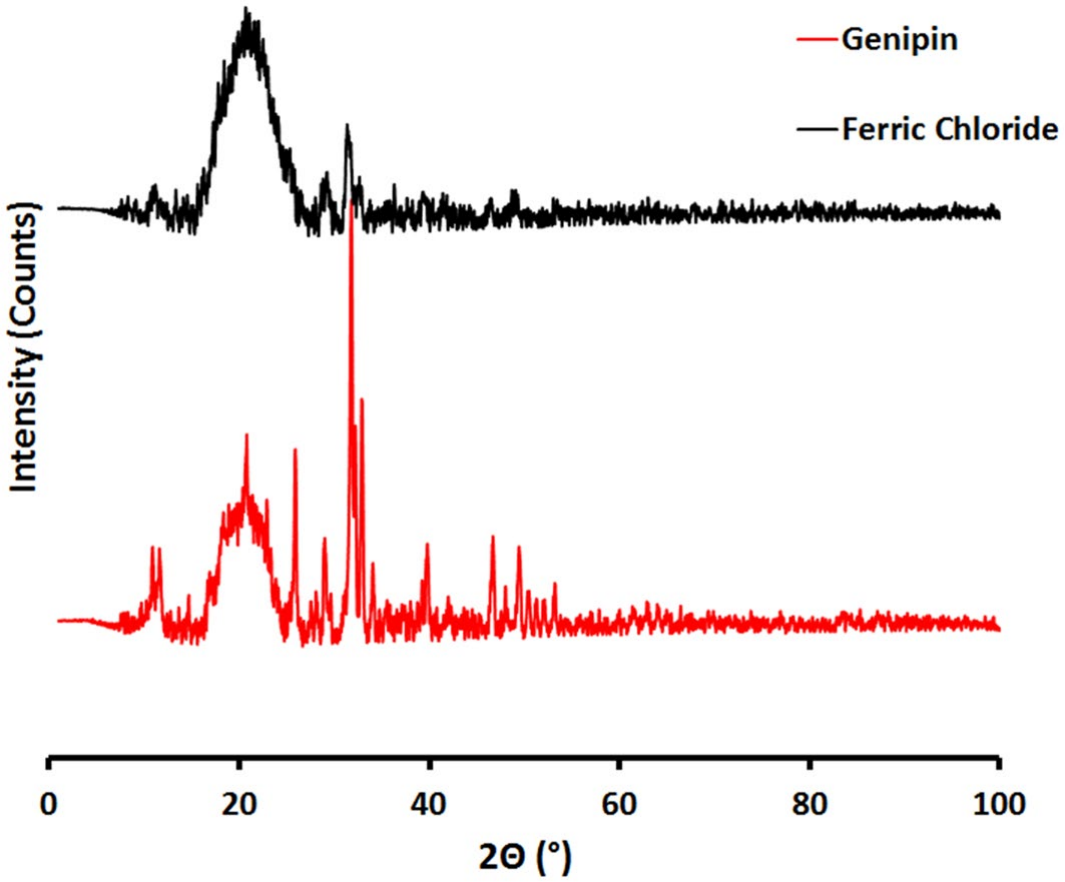
X-ray diffraction (XRD) analysis of the prepared GA/Ch/nHA hydrogels prepared using FeCl_3_ and Genipin cross-linkers.

The FeCl_3_ cross-linked hydrogel (Fig. 9) reveals a broad peak in the range of 15°–25° (the characteristic broad peaks of GA/chitosan are around 20° and 24°, respectively), which is in full agreement with the assumption that chitosan and GA are partially crystalline polysaccharides, which contain some crystalline forms embedded in the amorphous region. The latter peak was flattened in the Genipin cross-linked hydrogel group (Fig. 9). Thus, the Genipin cross-linked hydrogel had a lower crystallinity index than the FeCl_3_ cross-linked hydrogel; Cross-linking introduces a defect in the crystalline structure of polymers, and thus, results in a reduced crystallinity. The increased density of cross-linking results in a slower crystallisation process and a reduction in the final crystallinity of the networks. It can therefore be concluded that cross-linking with Genipin resulted in an increase in the amorphousness of the networks^1^. Also, the Genipin cross-linked hydrogel revealed a sharp peak at 2θ = 30°, which is consistent with the crystallinity of the nHA^2^.

#### Water absorption ASTM D570

**Figure 10:**
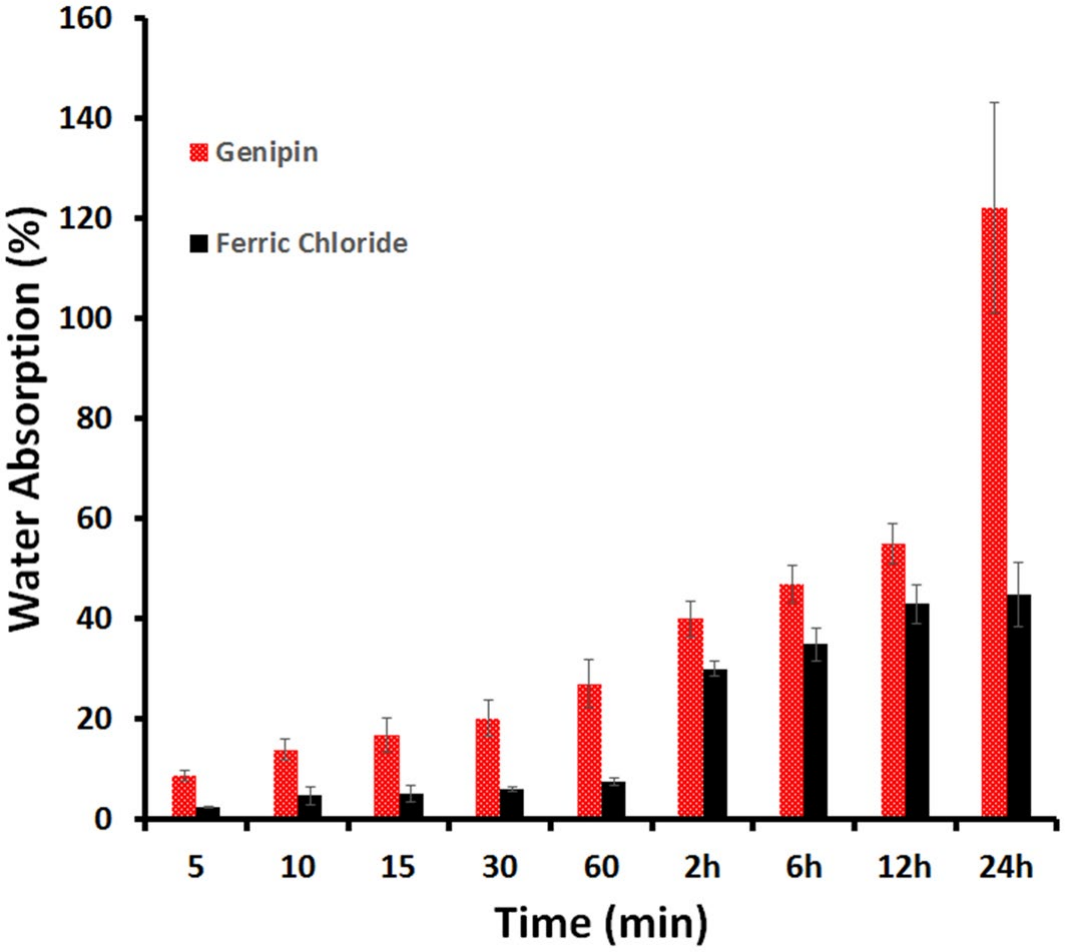
Water absorption versus time for the GA/Ch/nHA hydrogels prepared using FeCl_3_ and Genipin cross-linkers.

The water absorption behavior of the hydrogels is significant during in vitro cell culture studies. The swelling of the hydrogels allows for the absorption of body fluids as well as the transfer of cell nutrients and metabolites within them. Swelling also increases the internal surface area accessible for cell infusion and adhesion by expanding the pore size of the hydrogels. As shown in Fig. 10, the swelling ratio of the genipin cross-linked GA/Ch/nHA hydrogels was substantially higher than that of the hydrogels cross-linked with the FeCl_3_ especially at 24 h showing an average of 122 ± 21.09% versus 45 ± 6.51% respectively with a 77% increase in the water absorption. This can be attributed to genipin increasing the hydrophilicity of the network^1^, as well as increasing the porosity as clearly noticed in the SEM imaging. Also, the swelling ratio of the hydrogels cross-linked with the FeCl_3_ were equilibrated after 12 h as shown in Fig. 10, while that of the genipin group equilibrated after 24 h. This could be because genipin cross- linker is more hydrophilic than the FeCl_3_ cross-linker and can bind more water. On the whole, the hydrophilicity of hydrogels enables the absorption of body fluid, which is mostly water, and which is necessary for nutrient and metabolite diffusion^2^.

#### In vitro degradation behavior

**Figure 11:**
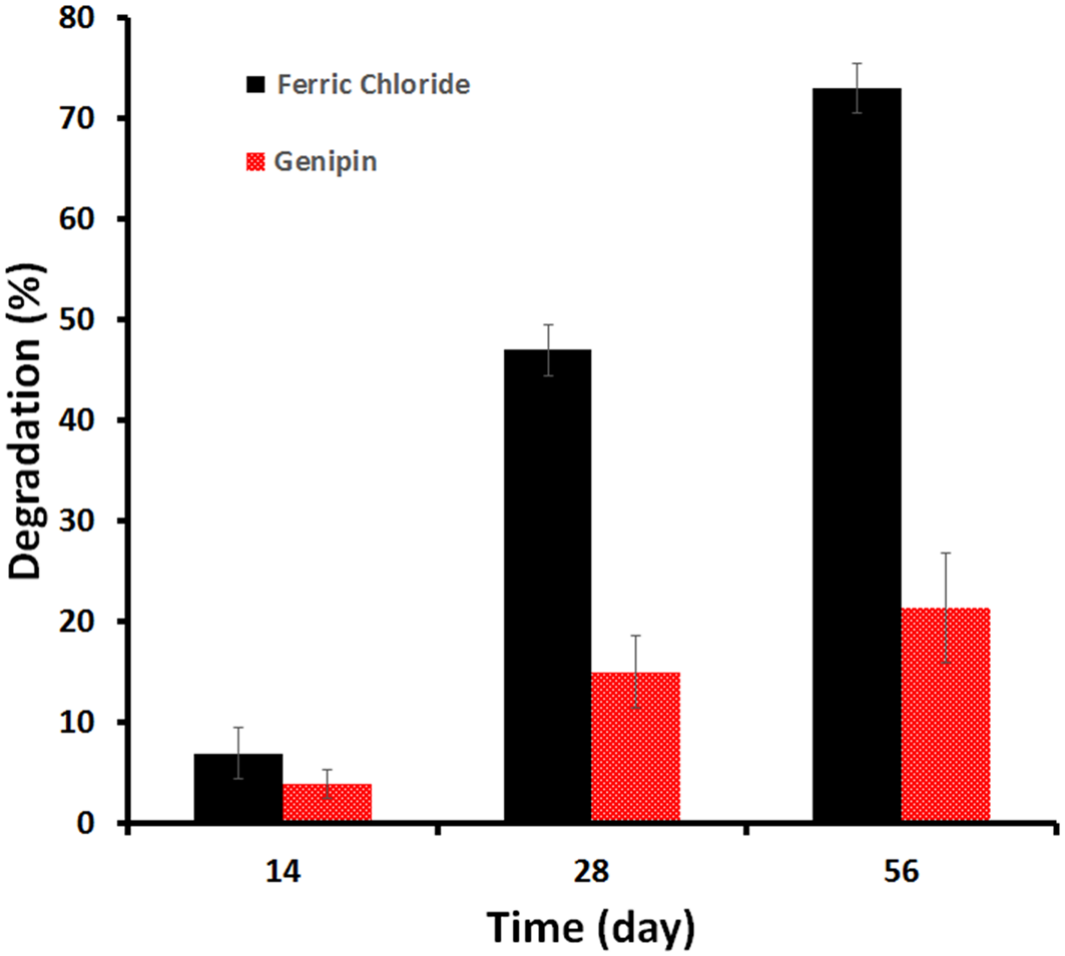
In vitro degradation of the GA/Ch/nHA hydrogels prepared using FeCl_3_ and Genipin cross-linkers in phosphate buffered saline (PBS) at 37 °C.

The physiological stability of composite hydrogels is crucial during bone regeneration; ideally, a scaffold should be degradable by endogenous enzymes or hydrolysis, synchronizing with new bone ingrowth to make enough space for new bone formation^34^. The degradation of the FeCl_3_ and genipin cross-linked hydrogels in PBS solution was studied for 2 months. As shown in Fig. 11, the highest average degradation rate of 73 ± 2.5% was observed in the FeCl_3_ cross-linked hydrogel after 56 days, while an average of 21.4 ± 5.4% degradation rate was observed in the genipin cross-linked hydrogels at the same time interval. This may be due to the increased cross-linking of the genipin nanocomposite hydrogels forming a more stable and compact hydrogel (scaffold) structure as confirmed by previous studies^1^. The hydrogel’s relatively slow biodegradation rate could be valuable in maintaining the hydrogels’ mechanical integrity while allowing enough time for bone growth to occur in the implant^2^.

#### Apatite-forming ability

**Figure 12:**
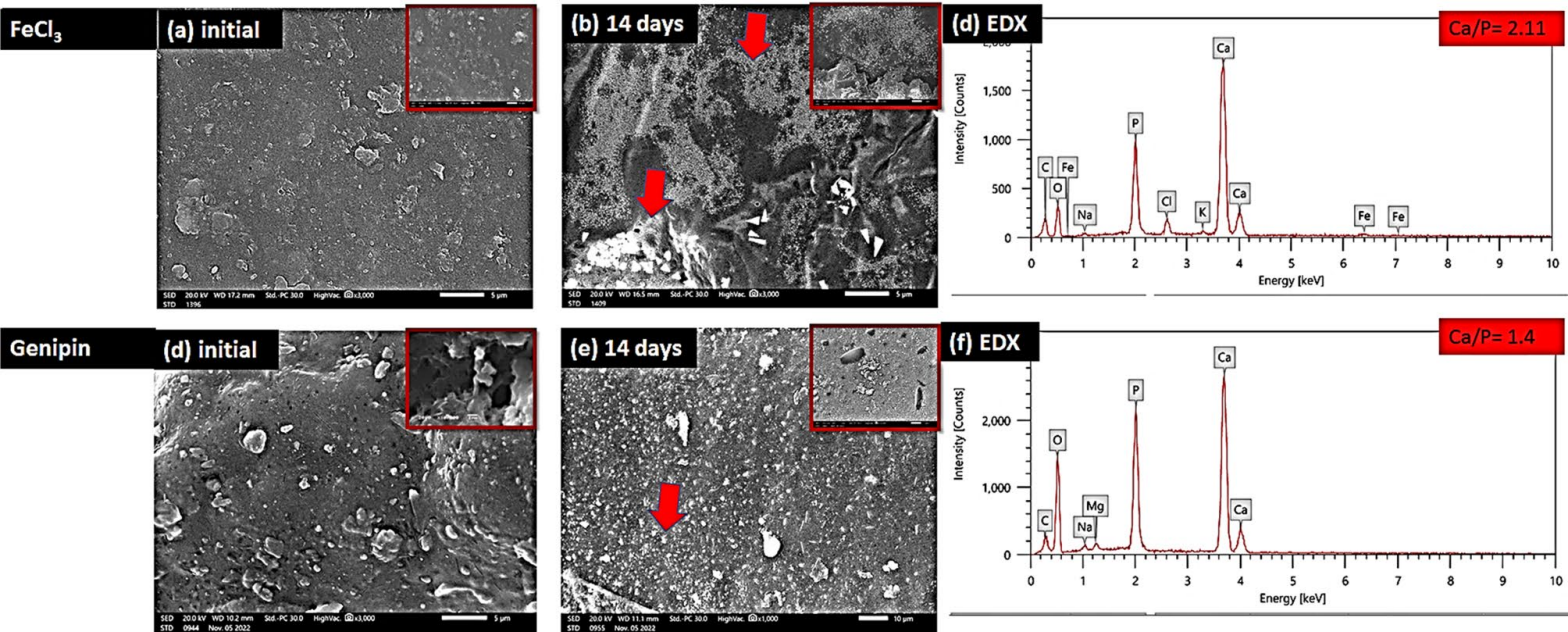
SEM images and EDX analysis of the GA/Ch/nHA hydrogels prepared using FeCl_3_ and Genipin cross-linkers after 14 days of incubation in simulated body fluid (SBF) with arrows pointing to some of the apatite particles.

All samples of the hydrogels developed a layer of spherical apatite particles with sizes ranging from about 1 µm to nanosized after 14 days of exposure to the simulated bodily fluid (SBF), as illustrated in Fig. 12. The surface layer showed extensive grain agglomeration. The particles were either found on the surface of granules that had already been created or as agglomerates of new phase particles that are loosely packed, with sinuous pores of various depths and with diameters distinguishable between them. Also, it was found that the hydrogels prepared using FeCl_3_ cross-linker had a higher concentration of apatite particles on their surface as compared to the Genipin cross-linked group after 14 days of incubation in simulated body fluid (SBF). This agrees with the findings of Koh M. et al.^35^, that stated that increasing the cross-linking reactions reduces the number of carboxyl groups that are thought to act as functional groups to induce heterogeneous nucleation of hydroxyapatite particles after soaking in SBF^35^. The significance of apatite formation on the surface of the hydrogel as reported by previous studies, has shown to promote osteoblast proliferation and differentiation. Furthermore, the ability to shape apatite can also be used to assess osteogenic bioactivity in vitro^36^.

#### Digital planar tomography

**Figure 13:**
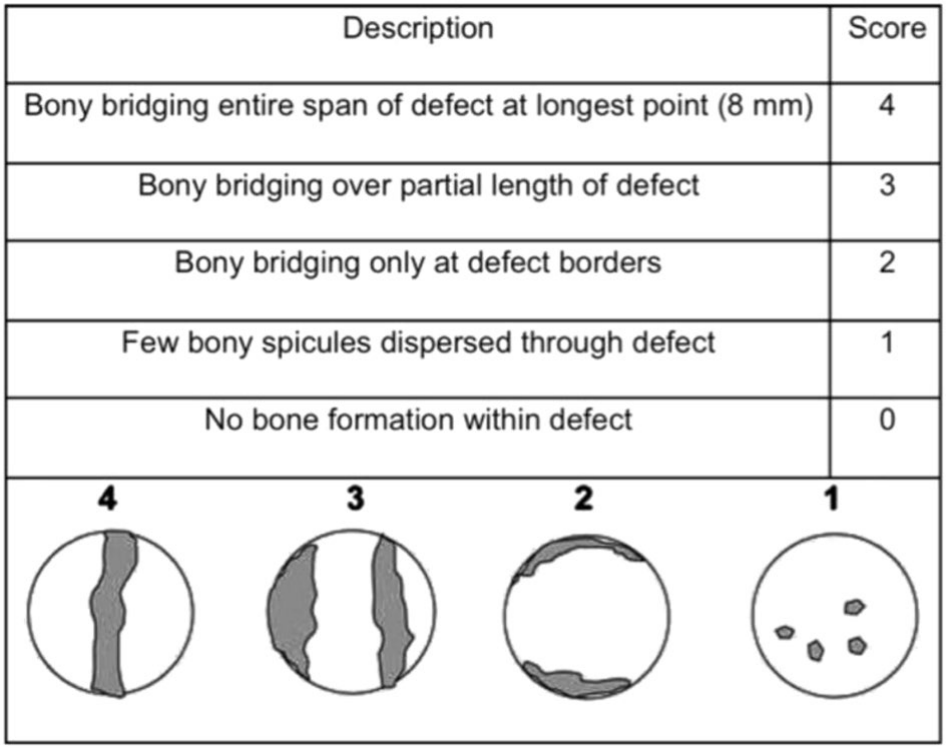
Scoring guide for the extent of bony bridging and union. The gray areas in the circles represent mineralized tissue formed within the defect, which can be used for planar radiographs or micro CT data sets^38^.

**Figure 14:**
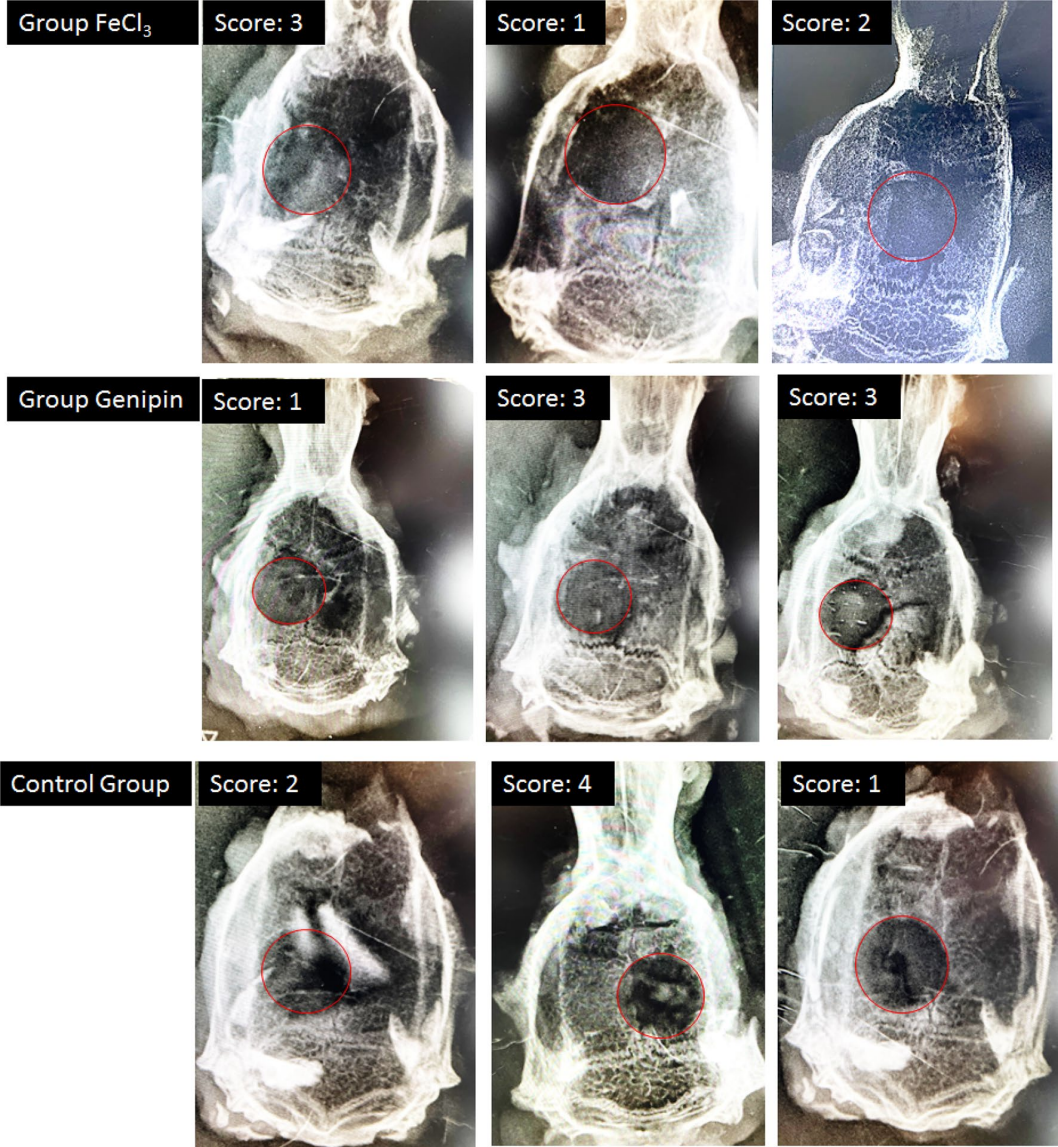
Ex vivo planar digital radiography of the three groups at 4 weeks; the GA/Ch/nHA hydrogels prepared using FeCl_3_: group 1, and Genipin cross-linker: Group 2 and an empty defect as negative control is Group 3.

Four weeks post implantation, animals were euthanized and calvarial tissues were harvested for analysis. Ex vivo planar digital radiography (RVG 142) was employed to evaluate the status of bone healing and each planar radiograph was scored according to the scoring system described in Fig. 13^25^. The size of the remaining defect treated with Genipin cross-linked hydrogel was remarkably smaller than that of the FeCl_3_ cross-linked hydrogel group, representative images are shown in Fig. 14. The relative new bone growth scoring guide for the extent of bony bridging and union were extracted from the digital X-ray images using the scoring guide in Fig. 13. The normalization was based on the original 6 mm defect area (Fig. 14). The defects that were treated with FeCl_3_ cross-linked hydrogel had a score of 2 ± 1 while the Genipin cross-linked hydrogels and control groups had a score of 2.33 ± 1.15 respectively. This may be due to an increase in hydrophilicity of the hydrogel surfaces by the addition of genipin, which may promote cell adhesion and growth. Even though further in vivo studies are needed, these results provide evidence to support that for implantation, a stiffer gel with higher genipin content and good mechanical robustness would produce favorable cellular responses^37^.

#### Histopathological evaluation

**Figure 15:**
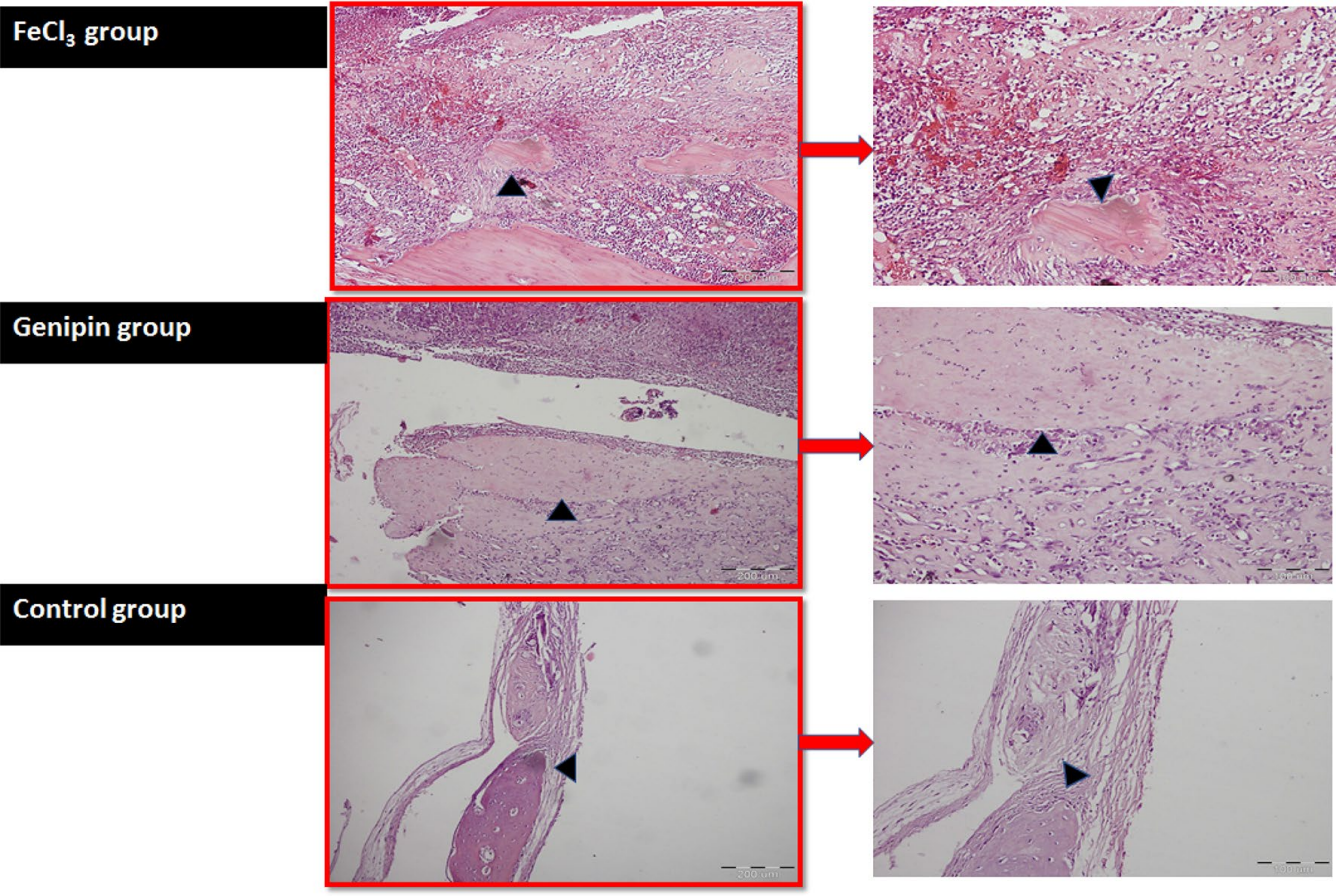
Histological micrographs (stained with H & E) at 4 weeks for the three groups; the GA/Ch/nHA hydrogels prepared using FeCl_3_; group 1, and Genipin cross-linker: Group 2 and an empty defect (control group) as negative control is Group 3 respectively, with arrowheads indicating the new bone.

**Figure 16:**
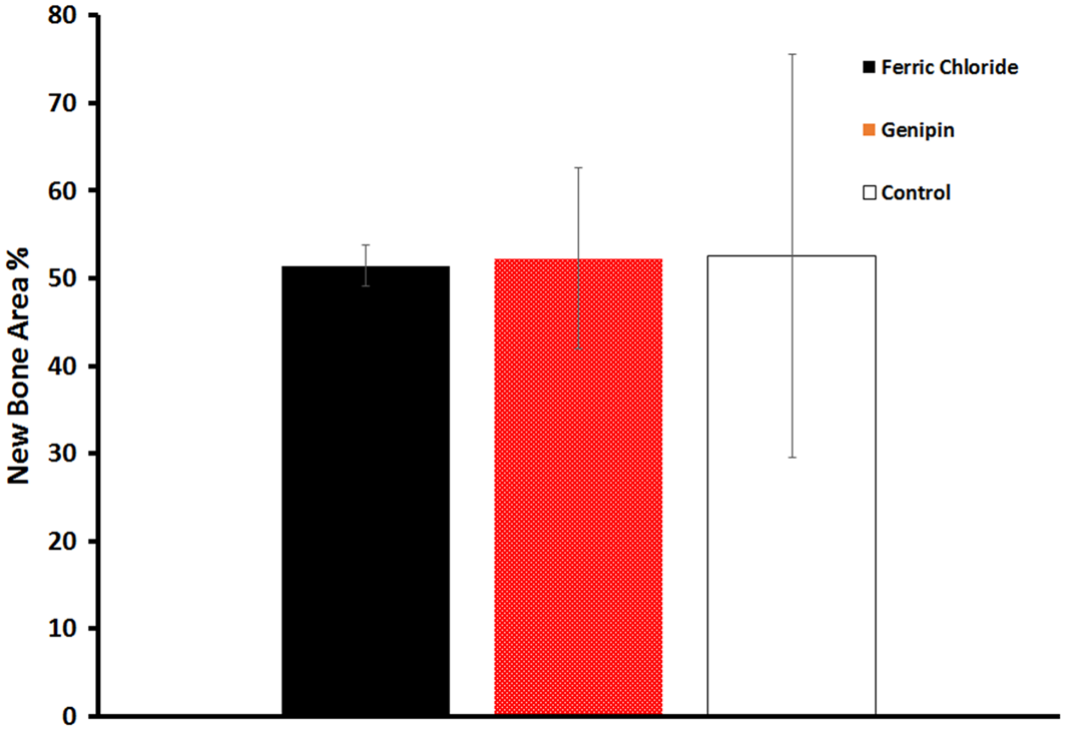
Histomorphometric quantitative analysis of new bone area (%) calculation of the GA/Ch/nHA hydrogels prepared using FeCl_3_ and Genipin cross-linkers, and an empty defect as negative control.

Histological examination using Hematoxylin and Eosin stain (H&E) was consistent with the planar digital radiography findings. All three groups revealed highly improved cell infiltration with thick granulation tissue formation composed of inflammatory cells and collagen-producing fibroblasts in the defect area with de novo bone formation (Fig. 15). Although the control group showed granulomatous tissue which may interfere with the bone healing process as shown in Fig. 15, the Genipin cross-linked hydrogel group revealed higher osteoid bone formation at 4 weeks as compared with the FeCl_3_ cross-linked hydrogel group. This may be due to the higher biocompatibility and increased porosity of the Genipin group as compared to the FeCl_3_ group which increases the cellular infiltration and subsequent bone formation^30^. Moreover, the histomorphometric quantitative analysis of the new bone area (%) (Fig. 16), revealed 51.43% ± 2.3 for the FeCl_3_ group; 52.27% ± 10.3 for the Genipin group; and 52.56% ± 22.9 for the control group respectively. These results are in agreement with the planar digital radiography results. In this context, the addition of genipin cross-linker enhanced the bone regeneration ability of the nanocomposite hydrogel over that of the FeCl_3_ cross-linker group^39^.

## Conclusion

In this work, two nanocomposite hydrogels were successfully prepared of unmodified Gum Arabic (GA), Chitosan (Ch), and natural nano-Hydroxyapatite (nHA) using an acrylic acid solvent and cross-linked with either physical (FeCl_3_) or chemical (genipin) cross-linkers. The comparison between some of the properties of these two nanocomposite hydrogels are shown in Table 1.

**Table 1:** Comparison between properties of the prepared GA/Ch/nHA hydrogels prepared using FeCl_3_ and Genipin cross-linkers.

Property	Genipin cross-linker	Ferric chloride cross-linker
Porosity	++	+
Compressive strength	++	+
Thermal stability	+	+
Hydrophilicity	++	+
In vitro degradation	+	++
Apatite forming ability	+	++

Using genipin cross-linker induced the corrugation of the scaffold surface and the emergence of more interconnected pores as revealed in the SEM images as compared to the FeCl_3_ cross-linked GA/Ch/nHA hydrogels. These pores could potentially encourage cell adhesion and aggregation as well as ingrowth along the pores of scaffolds.

Also, the Genipin cross-linked GA/Ch/nHA hydrogels increased the compressive strength from 10.2 to 19.2 MPa as compared to the FeCl_3_ cross-linked GA/Ch/nHA hydrogels, which is an 88% increase. Ultimately, success of a scaffold at its site of action is determined by its mechanical qualities particularly in the regeneration of hard tissues as bone.

Furthermore, using genipin cross-linker increased the hydrophilicity of the hydrogels over those cross-linked by FeCl_3_ as evident by the increased water absorption of 77% after 24 h. The hydrophilicity of the hydrogels enables the absorption of body fluid, which is mostly water, and which is necessary for nutrient and metabolite diffusion. However, the in-vitro degradation decreased significantly by 52% after 2 months in the Genipin cross-linked hydrogels as compared to the FeCl_3_ cross-linked hydrogels. However, the scaffold's slow biodegradation rate can be advantageous because it keeps the scaffold's mechanical integrity while giving the implant enough time for bone development.

In addition, the ex vivo planar digital radiography and histostopathological evaluation of the rat critical size calvarial defect both revealed new bone formation in both cross-linked hydrogel groups which confirms the bone regenerative potential of the two successfully prepared nanocomposite hydrogels.

## Data Availability

All data generated or analyzed during this study are included in this published article.
